# Analysis of Non-Compulsory Influenza and COVID-19 Vaccination among Polish Soldiers

**DOI:** 10.3390/ijerph20043304

**Published:** 2023-02-13

**Authors:** Ewelina Ejchman-Pac, Julian Wójtowicz, Magdalena Zawadzka

**Affiliations:** 1 Independent Department of Epidemiology, Military Institute of Hygiene and Epidemiology, 01-163 Warsaw, Poland; 2 Department of Medical Law Lodz, Medical University of Lodz Faculty of Health Sciences, 90-419 Lodz, Poland; 3 Department of Epidemiology and Public Health Lodz, Medical University of Lodz, 90-419 Lodz, Poland

**Keywords:** influenza, COVID-19, non-mandatory vaccination, army (soldiers)

## Abstract

The COVID-19 pandemic posed many challenges in epidemiology, health care, and vaccinology. Pharmaceutical and biotechnology companies had to develop effective vaccines as soon as possible in order to halt the spread of infection outbreaks and enable the start of the National Vaccination Program. Firstly, medical services and security services (the army, fire brigade, and police), i.e., those most involved in the fight against the effects of the COVID-19 pandemic, were included in the aforementioned program. The presented publication analyzes the amount and type of vaccination against COVID-19 and influenza among Polish soldiers. Influenza, like COVID-19, is a viral disease that can vary in its course (from mild to acute and life-threatening). Both coronaviruses and influenza viruses are characterized by high genetic variability, resulting in the need for repeated vaccination during each autumn and winter season. Acquired data comes from the Central Register of Vaccination of Professional Soldiers. The collected material was statistically processed. The average level of the phenomenon was presented as a time series using a chronological average. In the analyzed period (December 2020–December 2021), the lowest vaccinations against COVID-19 were performed in December 2020, which is due to the schedule of the National Vaccination Program in Poland. In contrast, the highest number of vaccinations were administered between April and June 2021, or approximately 70.5% of all vaccines administered. In the case of influenza, there is a clear increase in the number of vaccinations during the autumn and winter seasons, which coincides with peaks in disease during these periods. Between August 2020 and January 2021, there is a noticeable increase in the number of flu injections given, nearly 50% compared to the previous period, which may be related to the simultaneous persistence of the COVID-19 pandemic and greater attention to one’s own health. Non-mandatory vaccination is an important point in the vaccination schedule for soldiers. Numerous public campaigns combating misinformation and raising awareness of the need for immunization will help convince even more people, not only among soldiers but also the civilian population, to vaccinate.

## 1. Introduction

The COVID-19 pandemic is still an ongoing epidemiological issue worldwide. The only effective protection against the severe course of this illness is vaccination. Many biotechnological and pharmaceutical companies continuously work on vaccines against new variants of the coronavirus. In Poland, three vaccine doses (the first and two booster doses) are recommended. Currently, Pfizer and BioNTech mRNA vaccines Comirnaty (BNT162b2), Comirnaty Original/Omicron BA.1. and Comirnaty Original/Omicron BA.4/BA.5., Moderna-Spikevax (mRNA 1273), and Spikevax Bivalent Original/Omicron BA.1. have been approved in Poland as well as vector vaccines from Astra Zeneca-Vaxzevria (ChAdOx1 nCoV-19), the Johnson & Johnson Pharmaceutical Companies of Johnson & Johnson-Jcovden (COVID-19 Vaccine Janssen) (Ad26.COV.2-S), and a protein vaccine from Novavax-Nuvaxovid (NVX-CoV2373) [1]. No vaccine protects 100% of all those vaccinated; however, vaccination can effectively increase and improve protection against COVID-19 recurrence [2,3].

The European Medicines Agency is also in the process of authorizing two further vaccines against COVID-19: a protein vaccine with adjuvant Vidprevtyn (Sanofi Pasteur) and an inactivated vaccine with two adjuvants, VLA2001 (Valneva). At the same time, preliminary step-by-step evaluations (rolling review) of other COVID-19 vaccines administered worldwide—Sputnik V vector vaccine (Gam-COVID-Vac) (Gamaleya Institute), inactivated vaccine (Vero Cell) with adjuvant Sinovac (step-by-step procedure as of 04/05/2021), and COVID-19 vaccine HIPRA inactivated vaccine (PHH-1V) (HIPRA Human Health S.L.U.) [2]—are ongoing. Marketing authorization of vaccines during the pandemic is the responsibility of the European Commission, following a positive recommendation from the Committee for Medicinal Products for Human Use (CHMP) within the European Medicines Agency (EMA) [4].

In Poland, the COVID-19 vaccination schedule is defined by the National Vaccination Program. As early as 20 days after the Polish government adopted the above mentioned document, a decree was published by the Minister of Health on the COVID-19 prevention method, introducing vaccination as a method of counteracting the epidemic [5]. It should be noted that this act violates the principle of ‘lex retro non agit’, as its content implies that it applies retroactively, from the date on which the vaccination campaign was launched. The National Vaccination Program should also have been promulgated in the form of a decree by the Council of Ministers to be legally established. However, neither the rules adopted in this program nor its content as of today constitute universally binding law. The program adopted by resolution—not decree—of the Council of Ministers is of an internal nature and is binding only on organizational units subordinate to the Council of Ministers [6]. The first vaccine against COVID-19 administered in Poland was on 27 December 2020. From that moment, the activities indicated in the National Vaccination Program began. Professional soldiers, along with medical personnel, were one of the first groups covered by this program. This is due to the fact that the Polish Army was actively involved in combating the effects of the pandemic and providing assistance to those in need during the ‘lockdown’. It should be mentioned that the COVID-19 vaccination is not mandatory but is recommended [3].

Influenza is a viral disease of the upper respiratory tract. It, like COVID-19, can be distinguished by a course that necessitates hospitalization of the patient as well as lengthy and costly treatment. Fatal cases are also reported each year. Seniors are a group particularly vulnerable to complications from influenza; hence, it is recommended that they are vaccinated regularly [7]. However, soldiers, posted in different regions of the world and responsible for the security of the country, should also have access to effective and safe vaccines against diseases that threaten them, including influenza.

To meet the need to curb seasonal flu epidemics and flu-like infections, many pharmaceutical companies are developing vaccines based on the latest virus variant (from the previous season). Due to the rapid change of influenza virus antigens and the rapid decline of post-vaccination immunity, flu vaccination should be performed annually. Influenza vaccination does not protect 100% against the disease, but it will make the course of the disease milder even if it occurs [7].

Moreover, it should be recalled that the obligation of persons residing on the territory of the Republic of Poland to undergo immunization within the framework of the Public Health Vaccination Program may only result from legal regulations. This obligation may be abolished, limited, or extended to other vaccinations only by the legislator and the Minister of Health (within the scope of the authorization to determine the list of infectious diseases covered by the obligation of protective vaccinations and people or groups obliged to undergo obligatory protective vaccinations). However, to date, mandatory vaccination of soldiers against COVID-19 or influenza has not been introduced in any legal act [6].

These are recommended vaccinations and may be required when directed to perform certain tasks within the service; however, the lack of such vaccinations can in no way be a reason to draw any official or disciplinary consequences against a soldier. It is therefore surprising to find the condition of vaccination for COVID-19 for candidates for military service adopted in the guidelines of the Chief of the General Staff currently in force, to which the Ombudsman draws attention by raising the possibility of a legislative initiative in this regard, as well as a formal violation of the provisions of the Act on Universal Obligation to Defend in force in November 2021 (which only defines the rules for submitting medical and psychological examinations and immunizations to soldiers sent to periodic military service outside the borders of the state; however, from this act does not derive in any respect the obligation of immunization for soldiers), and all normative acts (including decisions, guidelines, recommendations, communiqués or recommendations of the Ministry of Foreign Affairs) are in contradiction with the rule of law (including Article 7 of the Constitution) [8,9].

The aim of this study is to analyze the number of selected recommended, non-compulsory vaccinations, i.e., against influenza and COVID-19, among Polish soldiers during the period 2018–2021.

## 2. Methods

The analysis was based on data from the Central Register of Vaccination of Professional Soldiers maintained in the System of Military Records (SEW on-line), made available by the Centre for Epidemic Response of the Armed Forces (CRE SZ RP), concerning the number of influenza and COVID-19 vaccinations performed among Polish soldiers in 2018–2021. The collected material was statistically processed using Excel and Statistica 13.1 tools. The frequency distribution of the analyzed variables was presented graphically. Seasonality indices determined on the basis of the chronological average were used to assess trends in influenza incidence over the analyzed period. These indicators were calculated on the basis of the empirical values of the time series and the series smoothed by the numerical method of chronological averages, which were determined using the formula:

$${\bar{y}}_{ch}=\frac{\frac{1}{2}y_{1}+y_{2}+\ldots +y_{n-1}+\frac{1}{2}y_{n}}{n-1}$$

The equation of the trend line, a rectilinear function describing changes in the phenomenon over time, is expressed by an equation of the form: *y* = *a* × *t* + *b*, where *a* and *b* were expressed using the following formula:

$$a=\frac{\sum_{i=1}^{n}\left(t_{i}-\bar{t}\right)\times \left(y_{i}-\bar{y}\right)}{\sum_{i=1}^{n}{\left(t_{1}-\bar{t}\right)}^{2}}$$

$$b=\bar{y}-a\times \bar{t}.$$

## 3. Results

The number of COVID-19 vaccination injections performed in a given period was strictly dependent on the epidemiological situation and the availability of vaccines.

**Figure 1:**
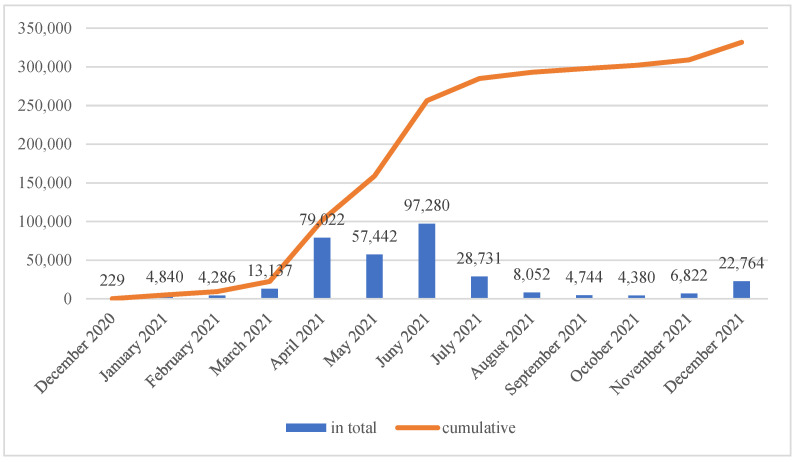
Number of anti-COVID-19 vaccinations performed among Polish soldiers (total and cumulative value).

It was noted that a total of 331,729 COVID-19 vaccines were administered between December 2020 and December 2021. The highest number of vaccinations were performed between April and June 2021, with a total of 233,744, representing 70.5% of all vaccines administered. The least number of vaccinations were performed in December 2020, which was related to the release of the vaccines and their availability only to health-related services (Figure 1).

**Figure 2:**
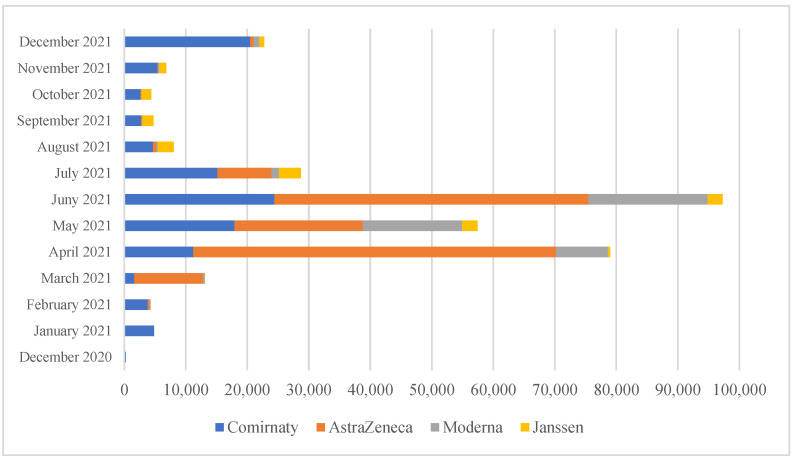
Number of anti-COVID-19 vaccinations administered to Polish soldiers by the vaccine manufacturer.

Uniformed services personnel were vaccinated with four different preparations. Almost every second person (i.e., 46.0%) was given a vector vaccine from AstraZeneca, a third, i.e., 34.7%, received an mRNA-based vaccine from Pfizer and BioNTech. One in five people were vaccinated with anti-COVID-19 preparation from Moderna (14.2%) or Johnson & Johnson (5.1%). Between March and June 2021, the most common vaccination was with an AstraZeneca formulation, with more than five times as many injections with this formulation compared to Pfizer and BioNTech in April 2021. On the other hand, from August to December 2021, several times more injections were given with the RNA vaccine (comirnaty) than with the vector vaccine (Figure 2).

A total of 41,374 influenza vaccinations were also performed during the analyzed period. The equation of the trend line has the following form: *y* = 119.69*x* + 275.55.

**Figure 3:**
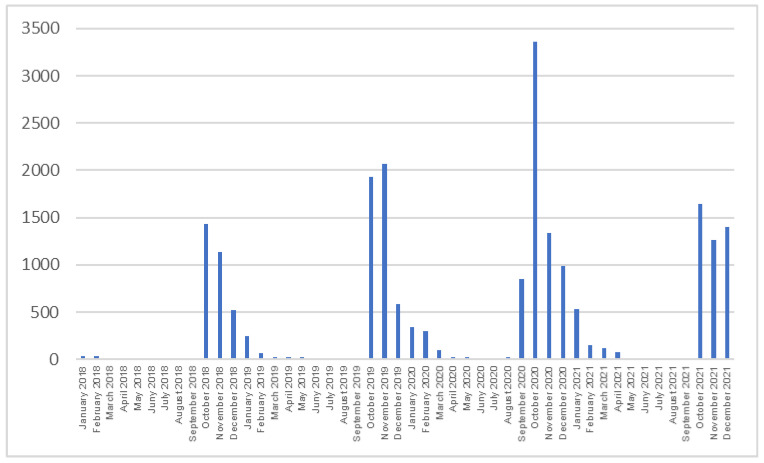
Number of anti-influenza vaccinations among Polish soldiers.

Flu vaccination shows a seasonality, falling in the autumn-winter period (Q4 of the year-Q1 of the year). In the period from August 2020 to January 2021, there is a noticeable increase in the number of influenza injections, nearly 50% compared to the previous period, which may be related to the simultaneous persistence of the COVID-19 pandemic and greater attention to one’s own health. Almost every year, the month of October is characterized by the highest number of vaccinations performed. In October 2020, the number of injections performed accounted for 45.2% of all vaccinations performed during the autumn-winter period 2020/2021 (Figure 3). During the period analyzed in this publication, Polish soldiers were vaccinated with seven types of vaccine, i.e., Vaxigrip tetra, Influvac, Vaxigrip, Afluria quaddrivalent, Flucelvax, Fluarix tetra, and Idflu 9. In the first two seasons, mainly the Influvac vaccine was administered; in the following two seasons, the Vaxigrip tetra was administered. This depended on the current availability of the preparations on the market.

**Figure 4:**
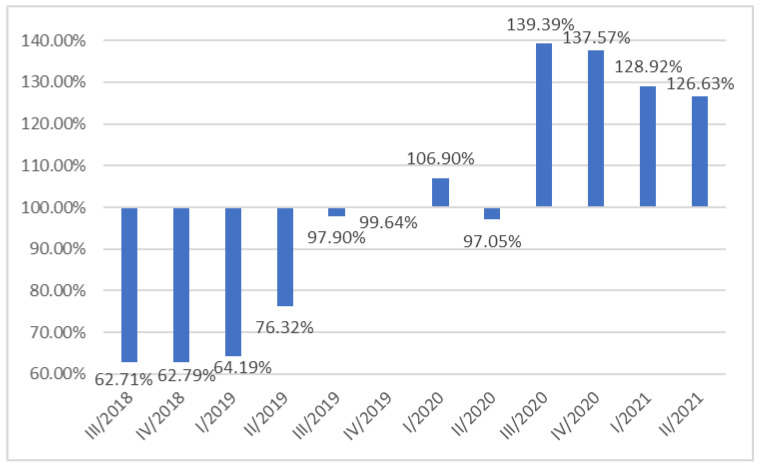
Seasonality of flu vaccination among Polish soldiers.

In Q4 2018 and Q1 2019, due to seasonal fluctuations, there were approximately 30% fewer influenza vaccinations performed than on an annual average basis. On the other hand, in Q3 and Q4 2020, as a result of seasonal fluctuations, nearly 40% more vaccinations were performed than on an annual average basis, and in Q1 and Q2 2021, nearly 30% more (Figure 4).

## 4. Discussion

An essential element of the combat value of the army is the anti-epidemic protection of soldiers, and the measures taken for this purpose, among them protective vaccinations, are aimed at creating safe working and service conditions. Polish military vaccination schedules are developed by the Operational Command of the Polish Armed Forces (DORSZ), taking into account the specific nature of the service, epidemiological threats, and the basis of the calendar of currently applicable preventive vaccinations. In Poland, there is a data register in which soldiers’ vaccinations are recorded (the Central Register of Vaccinations of Professional Soldiers). Supervision over the implementation of vaccinations is exercised by the authorities of the Military Sanitary Inspection and the Military Pharmaceutical Inspection. However, the people directly responsible are the commanders [10,11].

Both influenza and COVID-19 are viral infections that can have a dangerous course. Vaccinations against them are among the optional but recommended ones for members of the military. Soldiers in Poland, like other professional groups, were included in the national vaccination program against COVID-19, but no additional recommendations were developed for them. The current regulation does not provide any guidelines for vaccination against COVID-19, and for vaccination against pandemic influenza, it refers straight to WHO recommendations.

An interesting aspect of COVID-19 vaccination among the Israeli army is described by Tomer et al. They analyzed the difference between the declared willingness to receive the vaccine and the actual vaccination rate of Israeli soldiers. Participants were divided into three groups. The first attended a lecture prior to vaccination where the results of Pfizer’s vaccine clinical trials were presented, while the second benefited from individually scheduled appointments with a healthcare professional. The third, on the other hand, was able to obtain all the information from the doctor in the unit prior to vaccination. The findings presented in the publication demonstrate that social campaigns promoting vaccination contribute to greater awareness and help combat misinformation [12]. A similar study was conducted on a group of members of the US Air Force by Li et al.; they prepared a presentation on COVID-19 vaccination for the participants, followed by a questionnaire in which those recruited for the study had to assess how the information given during the lecture helped convince those who were hesitant or had negative attitudes to be vaccinated. On the basis of the survey, the authors conclude, that the main reason for reluctance to vaccinate is the under-reporting and spreading of misinformation about vaccination by anti-vaccination currents (or anti-vaxxers). An important factor in changing attitudes was trust in those providing data on the safety and efficacy of vaccination [13]. In their publication, Beymer et al. presented the results of a survey conducted in two US divisions. The first part of the survey concerned participants’ personal opinions on the safety and effectiveness of the COVID-19 vaccination and their plans to undergo vaccinations, while the second part concerned supervisors’ attitudes towards vaccinations. The percentage vaccinated was measured at two time points. Soldiers who identified vaccination in the survey as an effective method to combat a pandemic were more likely to undergo vaccination if given the opportunity. An interesting finding from the analysis is the attitude of the superiors towards health prevention. Soldiers whose command spoke positively about vaccination were more likely to undergo vaccinations [14]. In our study, soldiers did not participate in vaccination promotion campaigns; however, vaccination rates are high, with 233,744 vaccinations between April and June 2021, representing 70.5% of all vaccines administered in 2021.

The second optional vaccination that soldiers undergo is the vaccination against the influenza virus. Its high genetic variability leads to the development of dangerous strains that cause pandemics every few decades. The influenza virus, in its many variants, has contributed to the deaths of millions of people. In the 20th century, there were three such pandemics: in 1918–1919 (Spanish flu), 1957, and 1968. The desire to acquire immunity and counteract both the immediate effects of the disease and its complications seems to be a strong motivation for acquiring immunity through vaccination. However, this makes it necessary to vaccinate every year with a preparation developed from the virus identified in the previous season [15].

The National Institute of Public Health PZH-National Research Institute (NIZP PZH-PIB) in Poland pointed out that during the COVID-19 pandemic it is worth vaccinating against influenza for the following reasons: the occurrence of influenza and influenza-like infections together with COVID-19, the risk group for severe COVID-19 overlaps with the severe influenza group, overloaded health services due to the pandemic, which means difficult access to doctors, and people vaccinated against influenza are more resistant to other viral infections. Experts recommend simultaneous vaccination against COVID-19 and influenza, preferably before the start of the autumn/winter season [16]. This is because co-infection can have very serious health consequences for the patient, including death [17]. Despite the recommendations, according to NIZP PZH-PIB data, only 4% of the population in Poland is vaccinated against influenza and about 59% against COVID-19 [16].

The topic of vaccination among soldiers is also taken up by Arnold et al. in their publication, where they analyze the vaccination rate of members of the German army (both mandatory and recommended vaccinations). The two groups into which the participants were divided were analyzed for 12 months. The study group received individual notifications by email to be vaccinated or to receive a vaccination booster. The control group received verbal notification from the person responsible for implementing the vaccinations. The results of the analysis, conducted at the beginning of the study, indicate a low influenza vaccination rate in both groups (50.5% for the study group vs. 49.1% for the control group). After the end of the study, there was a slight increase in the vaccination rate among participants (63.3% for the study group vs. 64.7% for the control group). Nevertheless, this is still a low rate compared to the mandatory vaccination rates for, e.g., mumps, measles, and rubella (over 94%) [18].

Analyzing the period of the COVID-19 pandemic, Otieno and Rawlings highlighted an intervening fact. Studying a group of members of the British Army, they observed that the introduction of strictures at the time mentioned (social distance, wearing of masks, and partial work at home) resulted in a reduction in the number of reported cases of influenza and influenza-like infections among soldiers. The implication is that by maintaining safety and basic hygiene while in a large group of people, transmission of pathogens can be significantly reduced. A second finding described by the authors is the increase in the number of influenza vaccinations performed in the 2020/2021 season compared to the pre-pandemic season (16.3% vs. 18.4%) [19].

The COVID-19 pandemic has changed many people’s attitudes towards taking care of their health. This is also evident in the analysis of the authors’ own study, as nearly 40% more influenza vaccinations were performed in Q3 and Q4 2020 as a result of seasonal fluctuations compared to the annual average. In contrast, Marcus et al. surveyed a group of people living in rural areas to gauge their willingness to be vaccinated against influenza in the 2020/2021 season. Compared to the years preceding the pandemic, an increase in interest in influenza vaccination was observed. During the period analyzed by the authors, more than 88% of participants had been vaccinated against influenza, with the remaining 13.6% planning to be vaccinated in the near future. Unfortunately, 12.5% of respondents opted out of influenza vaccination due to the COVID-19 pandemic [20]. The topic of the impact of the COVID-19 pandemic on the perception of vaccination is also addressed by Nitzan et al. A survey of a group of Israeli soldiers shows that more than 50% of participants were determined to vaccinate against influenza. Of the participants who indicated in the survey that they were unlikely to plan to vaccinate (26% hesitated and 21% indicated ‘definitely not’), more than half declared good general health as the reason, and more than 36% claimed they had received too many doses of vaccination (mainly against COVID-19) during the 2021/2022 season. The authors add that unpublished reports indicate an increase in the number of flu vaccinations among the Israeli armed forces during the 2021/2022 season [21].

Similarly, in the authors’ own study, there was a noticeable increase in the number of influenza injections performed between August 2020 and January 2021, up nearly 50% compared to the preceding period, which may have been related to the concurrent duration of the COVID-19 pandemic and greater attention to one’s own health.

In summary, vaccination is one of the main means of prevention against infectious diseases. It is therefore extremely important to run public campaigns to encourage people to get vaccinated, change attitudes, and help combat misinformation. Soldiers, as individuals in different environmental conditions and forming large groups, should have access to up-to-date knowledge on vaccination and be kept informed about the availability of new preparations.

## 5. Conclusions

There is a need to raise awareness of the importance of immunization as an essential tool in the fight against infectious diseases. Regular vaccination of soldiers is essential to minimize the spread of infectious diseases and may have a positive impact on the effectiveness of ongoing military operations.

## Data Availability

Data presented in this study are available on request from CRESZ. Data is not publicly available due to military property.
